# Romo1-Derived Antimicrobial Peptide Is a New Antimicrobial Agent against Multidrug-Resistant Bacteria in a Murine Model of Sepsis

**DOI:** 10.1128/mBio.03258-19

**Published:** 2020-04-14

**Authors:** Hye-Ra Lee, Deok-gyun You, Hong Kyu Kim, Jang Wook Sohn, Min Ja Kim, Jong Kuk Park, Gi Young Lee, Young Do Yoo

**Affiliations:** a Laboratory of Molecular Cell Biology, Graduate School of Medicines, Korea University College of Medicine, Korea University, Seoul, Republic of Korea; b Department of Biosystems and Biotechnology, College of Life Sciences and Biotechnology, Korea University, Seoul, Republic of Korea; c Department of Surgery, Seoul National University College of Medicine, Seoul, Republic of Korea; d Division of Infectious Diseases, Department of Internal Medicine, Korea University College of Medicine, Seoul, Republic of Korea; e Division of Radiation Biomedical Research, Korea Institute of Radiological and Medical Sciences, Seoul, Republic of Korea; Korea Advanced Institute of Science and Technology

**Keywords:** AMPR-11, antimicrobial peptide, drug resistance, multidrug-resistant bacteria, peptide antibiotics, sepsis

## Abstract

To overcome increasing bacterial resistance to conventional antibiotics, many antimicrobial peptides (AMPs) derived from host defense proteins have been developed. However, there are considerable obstacles to their application to systemic infections because of their low bioavailability. In the present study, we developed an AMP derived from Romo1 (AMPR-11) that exhibits a broad spectrum of antimicrobial activity. AMPR-11 showed remarkable efficacy against sepsis-causing bacteria, including multidrug-resistant strains, with low toxicity in a murine model of sepsis after intravenous administration. It seems that AMPR-11 disrupts bacterial membranes by interacting with cardiolipin and lipid A. From the results of this study, we suggest that AMPR-11 is a new class of agent for overcoming low efficacy in the intravenous application of AMPs and is a promising candidate to overcome multidrug resistance.

## Abstract (precis)

Abuse of antibiotics often leads to increase of multidrug-resistant (MDR) bacteria, which threatens the life of human beings. To overcome threat of antibiotic resistance, scientists are developing a novel class of antibiotics, antimicrobial peptides, that can eradicate MDR bacteria. Unfortunately, these antibiotics have mainly been developed to cure bacterial skin infections rather than others, such as life-threatening sepsis. Major pharmaceutical companies have tried to develop antiseptic drugs; however, they have not been successful. Here, we report that AMPR-11, the antimicrobial peptide (AMP) derived from mitochondrial nonselective channel Romo1, has antimicrobial activity against Gram-positive and Gram-negative bacteria comprising many clinically isolated MDR strains. Moreover, AMPR-11 increased the survival rate in a murine model of sepsis caused by MDR bacteria. We propose that AMPR-11 could be a novel antiseptic drug candidate with a broad antimicrobial spectrum to overcome MDR bacterial infection.

## INTRODUCTION

Antibiotics are one of the most revolutionary medicines for human therapy and have decreased the mortality of patients from bacterial infections (1). However, antibiotic abuse has increased the emergence of antibiotic-resistant bacteria, now paradoxically threatening the lives of human beings (2). Indeed, a research charity called the “Wellcome Trust” warned that 10 million people would die in 2050 and that an additional consequence of antimicrobial resistance would be an increase in global economic burden (3). The most notorious bacteria that are susceptible to antibiotic resistance are collectively known as ESKAPE: Enterococcus faecium, Staphylococcus aureus, Klebsiella pneumoniae, Acinetobacter baumannii, Pseudomonas aeruginosa, and *Enterobacter* species (4). Since patients suffering from nosocomial infections with multidrug-resistant (MDR) Gram-negative bacteria have poor clinical outcomes, the World Health Organization (WHO) recently reported carbapenem-resistant A. baumannii (CRAB), carbapenem-resistant P. aeruginosa (CRPA), and *Enterobacteriaceae* as the highest priority, all of which are carbapenem-resistant Gram-negative bacteria. Compared to MDR Gram-positive bacteria (e.g., methicillin-resistant *S. aureus* [MRSA] or vancomycin-resistant *S. aureus* [VRSA]), which are considered to be of secondary priority, there are only a few options against carbapenem-resistant Gram-negative bacteria (5).

Although the development of chemical antibiotics in major pharmaceutical companies has decreased along with development of antibiotics because of lack of investment returns (6), scientists are developing a novel class of antibiotics, antimicrobial peptides (AMPs), to eradicate MDR bacteria (7). However, AMP developments have been focused on topical treatment for MDR bacteria causing skin and soft tissue infections (SSTIs) as a first indication (8, 9). The first AMP to reach clinical trial was Locilex, or pexiganan, a magainin analog isolated from the skin fluid of the African clawed frog and indicated for treatment of diabetic foot ulcer (DFI). However, its New Drug Application (NDA) was rejected by the U.S. Food and Drug Administration (FDA) in 1999 because of its low efficacy compared to chemical antibiotics (10). In 2004, it was reevaluated for DFI with two phase III trials (Onestep-1 and Onestep-2) with an enhanced formulation but did not meet the primary endpoints (11). Although many AMPs are currently under development, life-threatening infections such as MDR Gram-negative bacteremia have not been considered an indication of AMPs regardless of their high mortality. The lipopeptide colistin, a last-resort antibiotic that was discontinued in the 1980s because of neuro- and nephrotoxicity, is undergoing phase III trials for carbapenem-resistant Gram-negative bacteremia (12).

This situation becomes much more severe in septic patients. Sepsis is a life-threatening systemic inflammation caused by pathogens, mainly bacteria (13). Major pharmaceutical companies have tried to develop antiseptic drugs but have not been successful (14). Although recombinant human activated protein C (Drotrecogin alfa, Xigris) was developed by Eli Lilly for severe septic patients, it was withdrawn from the market in 2011 due to lack of efficacy compared to placebo in the PROWESS-SHOCK trial (15, 16). In that same year, orally available antimicrobial protein talactoferrin was evaluated for severe sepsis in the phase II/III OASIS trial, but the results were unsatisfactory (17).

According to sepsis guidelines, antibiotics should be administered within 1 h if patients show symptoms of sepsis, which means that neither the bacterial species nor the presence of MDR bacteria can be identified before antibiotic treatment (18). Therefore, an empirical antibiotic combination is intravenously administered as the initial step of sepsis treatment (19, 20). Considering that the ideal antimicrobials for sepsis should eradicate the bacteria regardless of species and presence of MDR, AMP could be the best antimicrobial candidate for sepsis caused by MDR bacteria. However, AMPs already developed and under development have a limitation for use in sepsis treatment due to lack of stability in blood (21).

It has been reported that expression of reactive oxygen species modulator 1 (Romo1) increased cellular reactive oxygen species (ROS) production and contributed to tumor progression (22–24). Recently, Romo1 was shown to function as a nonselective cation channel. This protein contains two transmembrane domains (TMDs), and its secondary TMD forms an amphipathic helical pore-forming domain (25). Because Romo1 is a nucleus-encoded mitochondrial protein and the membrane characteristics of mitochondria are similar to those of bacteria in terms of low membrane fluidity with a negatively charged surface, Romo1 might harbor antimicrobial activity against bacteria due to its pore-forming domain. In this study, we explored the possibility of the pore-forming domain of Romo1 as an AMP for treating sepsis caused by MDR bacteria and showed that AMP derived from Romo1 (AMPR-11) is a promising agent for treatment of sepsis caused by MDR bacteria.

## RESULTS

### Antimicrobial activity of Romo1 against intracellular invading bacteria.

**FIG 1:**
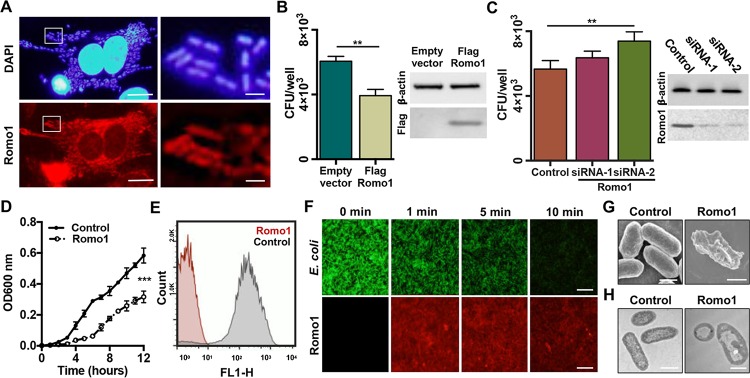
Antimicrobial activity of the Romo1 protein against EIEC. (A) Romo1 targeting the internalized EIEC in HeLa cells. HeLa cells were infected with EIEC and stained with anti-Romo1 antibody and DAPI. Scale bar, 10 μm (left) or 1 μm (right). (B and C) The number of internalized EIEC in Romo1 overexpressed (pcDNA3.1 or pcDNA3.1-Flag-Romo1 for 36 h) or knockdown (control siRNA, Romo1 siRNA-1, or Romo1 siRNA-2 for 48 h) cells. The expression level of Romo1 was confirmed by Western blotting. (D) Growth rate curves of EIEC incubated with 1 μg/ml of Romo1 protein. (E) GFP-expressing EIEC were incubated with 100 nM Romo1, and GFP efflux was measured by flow cytometry. (F) GFP-expressing EIEC were incubated with TAMRA-Romo1, and GFP efflux was visualized with fluorescence microscopy. Scale bar, 15 μm. (G and H) SEM and TEM images of EIEC incubated with Romo1. Sale bar, 1 μm. Data represent means ± the standard deviations (SD). **, *P* ≤ 0.01; ***, *P* ≤ 0.001 (by two-way analysis of variance [ANOVA]).

Because mitochondria and bacteria share an ancestor (26) and many intracellular bacteria can replicate within host cells (27), we posited that intracellular bacteria could be killed by nucleus-encoded proteins translocated into mitochondria, which harbor an amphipathic pore-forming domain, since amphipathic alpha-helical structures have antimicrobial activity. We focused on one of the mitochondrial nonselective ion channels, Romo1, which was recently shown to induce mitochondrial membrane permeabilization (25). Enteroinvasive Escherichia coli (EIEC), which has been reported to be internalized into the cells, was used in this experiment. We infected HeLa cells with EIEC and determined that Romo1 did target the internalized EIEC (Fig. 1A). The number of internalized EIEC in both Romo1-overexpressed and Romo1-knockdown cells was examined by flow cytometry and showed no difference (see Fig. S1 in the supplemental material). However, viability of the internalized EIEC was decreased in Romo1-overexpressed cells (Fig. 1B) and increased in Romo1-knockdown cells (Fig. 1C), indicating that Romo1 can eradicate the invaded bacteria.

10.1128/mBio.03258-19.1 FIG S1 Internalization of EIEC into HeLa cells. The number of invaded bacteria was examined with flow cytometry. Transfected cells were infected with EIEC to be stained with 50 μM CFDA. Download FIG S1, PDF file, 0.1 MB. Copyright © 2020 Lee et al. 2020 Lee et al. This content is distributed under the terms of the Creative Commons Attribution 4.0 International license.

Since Romo1 is not expressed in the E. coli expression system, it was chemically synthesized and was dissolved in 75% trifluoroethanol, an alpha-helix stabilizing agent, which has frequently been used as a solvent for alpha-helical transmembrane proteins (28). We applied Romo1 to EIEC directly and measured the bacterial growth by spectrophotometry. Romo1 treatment inhibited EIEC growth (Fig. 1D) and induced green fluorescent protein (GFP) efflux from GFP-expressing EIEC (Fig. 1E). To visualize Romo1 targeting the EIEC and the Romo1-induced GFP efflux, we administered 5-carboxytetramethylrhodamine (TAMRA)-labeled Romo1 to the GFP-expressing EIEC. TAMRA-Romo1 targeted the EIEC within 1 min, and GFP was released from EIEC in a time-dependent manner (Fig. 1F). Consistent with these results, bacterial membrane disruption was observed by scanning electron microscopy (SEM) (Fig. 1G) and transmission electron microscopy (TEM) (Fig. 1H). These results indicate that the Romo1 protein has antimicrobial activity through bacterial membrane permeabilization.

### K58-R78 region of Romo1 has antimicrobial activity.

**FIG 2:**
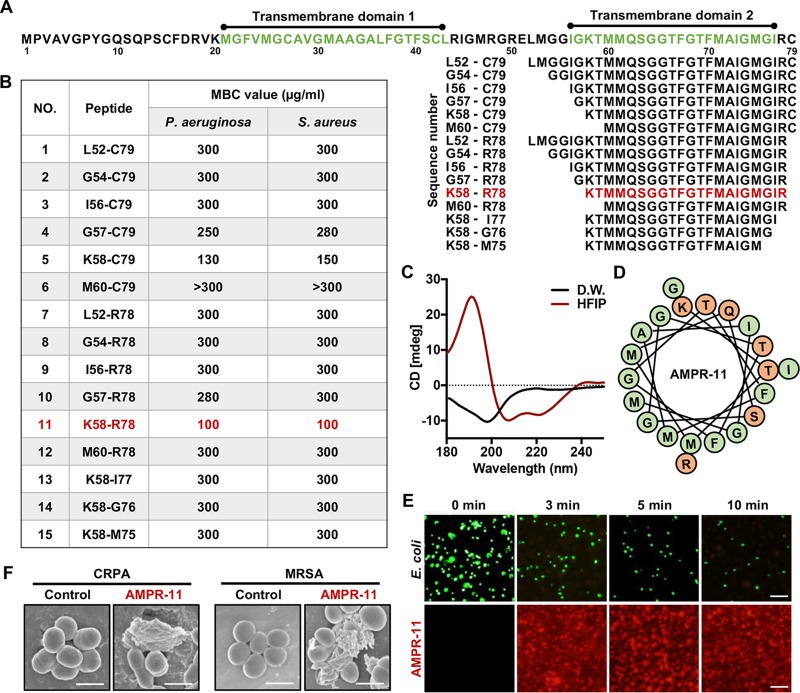
Antimicrobial activity of AMPR-11 (K58-R78) derived from Romo1. (A) Sequences of deletion mutants of Romo1. Green represents transmembrane domains (M21 to L43 and I56 to I77) by TMMTOP server. (B) Determination of the MBC of each deletion mutant to kill 10^5^ CFU of P. aeruginosa or S. aureus in 1 h. (C) Circular dichroism spectroscopy of AMPR-11. The black line represents the AMPR-11 structure in distilled water, and the red line represents the AMPR-11 structure in 50% HFIP. (D) Predicted alpha-helical wheel of AMPR-11. The illustration was recreated based on the Helical Wheel Projection server. Green, nonpolar amino acids; orange, polar amino acids. (E) GFP-expressing EIEC permeabilization by TAMRA-AMPR-11. Scale bar, 20 μm. (F) SEM images of CRPA or MRSA incubated with AMPR-11. Scale bar, 1 μm.

We next sought to determine the core region of Romo1 that harbors antimicrobial activity. Because Romo1 has an amphipathic alpha-helical transmembrane domain for pore formation, we expected that this region might be responsible for antimicrobial activity. To evaluate this hypothesis, we chemically synthesized 15 deletion mutants from their predicted amphipathic TMDs by sequential deletion (Fig. 2A). The deletion mutants were dissolved in water, and their antimicrobial activities were evaluated by using the minimum bactericidal concentration (MBC) test (29). The K58-R78 region showed the highest bactericidal activity among deletion mutants (Fig. 2B). This peptide was called AMP derived from Romo1-11 (AMPR-11). Next, we measured the antimicrobial activity of AMPR-11 against sepsis-causing bacteria, including their MDR strains. Interestingly, AMPR-11 showed a broad spectrum of antimicrobial activity against all bacteria tested in this study (see Table S1 in the supplemental material). We examined the secondary structure of AMPR-11 with circular dichroism. AMPR-11 formed a random coil structure in distilled water but formed an ordered conformation in 50% hexafluoro-2-propanol (HFIP), which has been used for implementing a membrane-mimic environment (30). This conformation showed one positive peak at 192 nm and two negative peaks at 208 to 210 nm and at 222 nm, indicating a predominantly alpha-helix structure (Fig. 2C and D). This implies that AMPR-11 with a random coil structure before membrane insertion forms an alpha-helical structure in the membrane environment. Similar to wild-type Romo1, AMPR-11 induced membrane permeabilization in GFP-expressing EIEC (Fig. 2E) and bacterial membrane disruption of CRPA or MRSA (Fig. 2F). These results indicate that the K58-R78 region of Romo1 is a new class of AMP derived from mitochondrial targeting protein with a broad spectrum of antimicrobial activity, including against MDR strains.

10.1128/mBio.03258-19.2 TABLE S1 MBC determination of AMPR-11 in multiple bacterial species. Download Table S1, DOCX file, 0.01 MB. Copyright © 2020 Lee et al. 2020 Lee et al. This content is distributed under the terms of the Creative Commons Attribution 4.0 International license.

### AMPR-11 induces membrane permeabilization by interacting with cardiolipin and/or lipid A.

**FIG 3:**
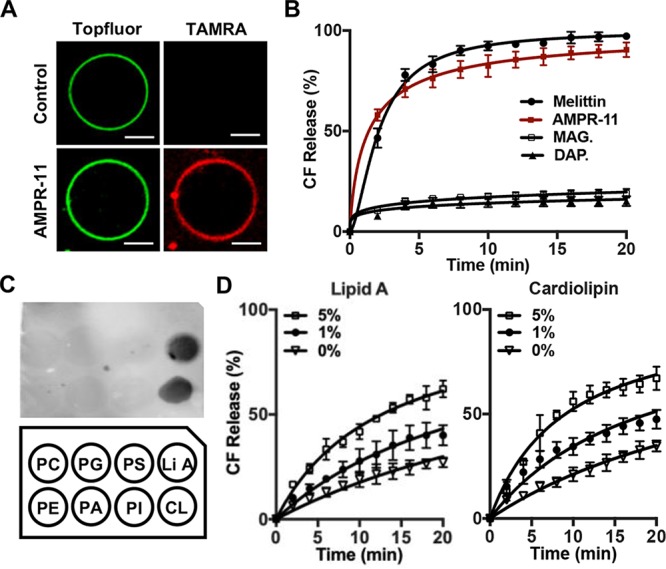
AMPR-11-induced liposome permeabilization and its lipid preference. (A) Targeting of AMPR-11 to bacterial membrane-mimic GUVs. GUVs were generated using E. coli total lipid extract with 0.5% TF-CHOL. GUVs were incubated with TAMRA-AMPR-11 for 1 min and analyzed with confocal microscopy. Scale bar, 10 μm. (B) CF-encapsulated LUVs permeabilization induced by AMPR-11. CF-LUVs were generated using E. coli total lipid extract and incubated with 1 μg/ml of melittin, magainin 2, daptomycin, or AMPR-11. CF release was monitored with a spectrophotometer. (C) Lipid preference of AMPR-11. Lipid strip containing each natural lipid (20 pmol) was incubated with TAMRA-AMPR-11. (D) CF-LUVs were generated using E. coli total lipid extract with additional lipid A or cardiolipin incubated with 500 ng of AMPR-11 for 20 min, and CF release was monitored with a spectrophotometer. Data represent means ± the SD. DAP., daptomycin; MAG., magainin 2.

Prior to examination of AMPR-11-induced membrane permeabilization, we tested whether AMPR-11 efficiently targets artificial giant unilamellar vesicles (GUVs) composed of E. coli total lipid extract. E. coli membrane-mimic GUVs were incubated with TAMRA-AMPR-11, and AMPR-11 targeted the GUVs (Fig. 3A). To evaluate liposome permeabilization, we generated carboxyfluorescein (CF)-encapsulated large unilamellar vesicles (CF-LUVs) composed of E. coli total lipid extract by extrusion, as previously described (31). AMPR-11 induced CF release from the CF-LUVs (Fig. 3B) to a similar extent as melittin (a pore-forming toxin of bee venom) and more than well-known antimicrobial peptides such as magainin 2 and daptomycin. To examine the lipid preference of AMPR-11, we performed a protein lipid overlay (PLO) assay. Interestingly, AMPR-11 specifically bound to lipid A and cardiolipin (CL) (Fig. 3C), bacterium-specific lipids, compared to other lipids used for the PLO assay. To confirm their importance in membrane permeabilization, lipid A and CL were increased in proportion in the CF-LUVs, and AMPR-11 induced CF release in a lipid A or CL concentration-dependent manner (Fig. 3D). These results indicate that lipid A and CL are possible targets of AMPR-11 to induce membrane permeabilization.

### Efficacy of AMPR-11 in a murine model of sepsis and toxicity.

**FIG 4:**
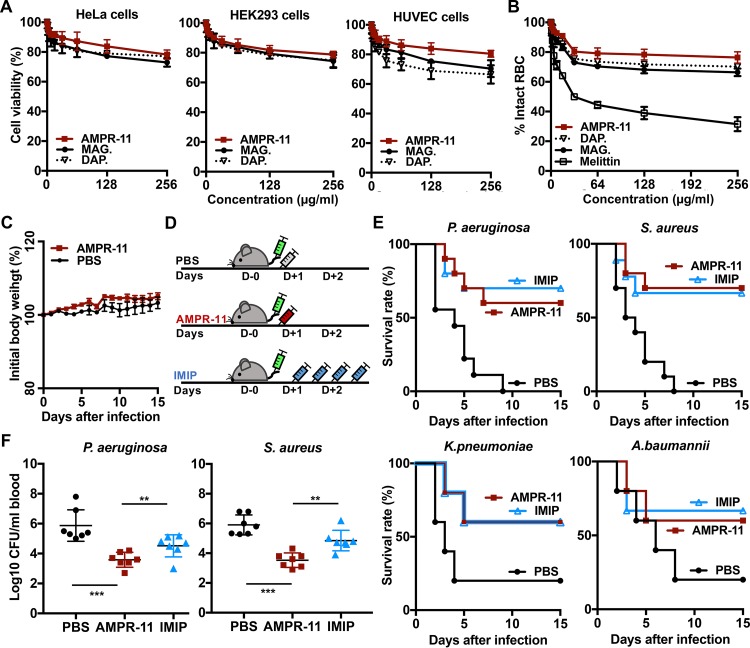
Antimicrobial activity of AMPR-11 in the murine model of sepsis. (A) Cell viability assay of mammalian cells (HeLa cells, HEK293 cells, and HUVECs) with daptomycin, magainin 2, or AMPR-11. Peptides from 0 to 256 μg/ml were incubated for 2 h and analyzed by an MTT assay. (B) Hemolysis assay of RBCs incubated with daptomycin, magainin 2, melittin, or AMPR-11. The percentage of hemolyzed RBCs was analyzed by spectrophotometer. (C) Body weight changes of C57BL/6 mice after intravenous administration of AMPR-11 (100 mg/kg) or PBS. (D) Experimental workflow of the sepsis model. (E) Survival rates in mice infected with P. aeruginosa, S. aureus, K. pneumoniae, or A. baumannii. Each group was treated with PBS, imipenem (10 mg/kg with intraperitoneal administration, four times q12h), or AMPR-11 (10 mg/kg with intravenous administration, single dose). Survival rates were calculated with 15 mice/group for K. pneumoniae and A. baumannii and 20 mice/group for P. aeruginosa and S. aureus. (F) Numbers of bacteria in mouse blood after 48 h of bacterial infection with or without AMPR-11 administration. Data represent means ± the SD. **, *P* ≤ 0.05; ***, *P* ≤ 0.001 (by two-way ANOVA). DAP., daptomycin; MAG., magainin 2; IMIP.; imipenem.

The toxicity of AMPR-11 in mammalian cells (HeLa cells, human embryonic kidney 293 [HEK293] cells, and human umbilical vein endothelial cells [HUVECs]) was examined prior to evaluation of its efficacy in a murine model of sepsis. AMPR-11 treatment exhibited less cytotoxicity (Fig. 4A) and hemolytic activity (Fig. 4B) than magainin 2 and daptomycin. To clarify its safety in mice, we intravenously administered a single dose (100 mg/kg) of AMPR-11 into a C57BL/6 mouse and tracked it for 15 days. There were no severe clinical signs such as death or weight loss compared to the control group (phosphate-buffered saline [PBS]) (Fig. 4C).

Next, we evaluated AMPR-11 efficacy in the murine model of sepsis. Mice were injected intravenously with P. aeruginosa (ATCC 27853), S. aureus (ATCC 29213), K. pneumoniae (ATCC 13883), or A. baumannii (ATCC 19606), and AMPR-11 was administered after 1 h of infection as described in the experimental workflow (Fig. 4D) (32). Imipenem was intraperitoneally administered four times every 12 h (q12h) as previously described, with minor modifications (33). Interestingly, a single dose (10 mg/kg) of AMPR-11, which was a 10-fold lower concentration when tested for *in vivo* toxicity, increased the survival rate by >60% in both Gram-positive bacteria (S. aureus) and Gram-negative bacteria (P. aeruginosa, K. pneumoniae, and A. baumannii) (Fig. 4E), with a decrease in the bacterial load in blood (Fig. 4F). These results indicate that a single dose (10 mg/kg) of AMPR-11 has similar efficacy to multiple doses of imipenem.

**FIG 5:**
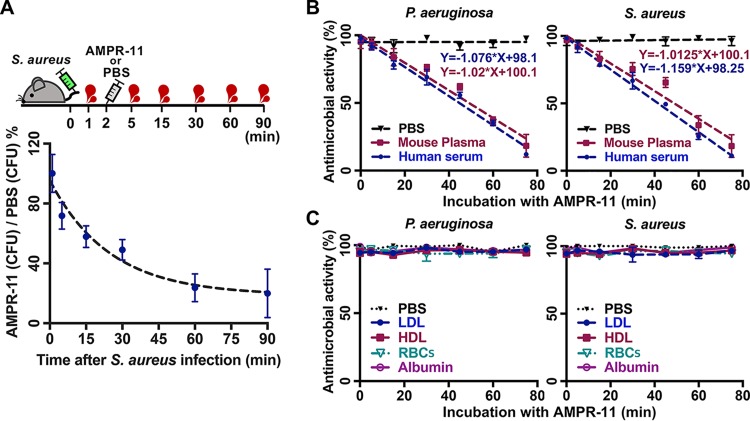
Effects of blood components on antimicrobial activity of AMPR-11. (A) Comparison of bacterial load in blood between PBS and AMPR-11. Mice were infected with S. aureus, and either AMPR-11 or PBS was subsequently administered. CFU of blood samples from the tail vein was calculated using the following formula: CFU of AMPR-11-administered mouse group / CFU of PBS group ×100 (%). (B and C) Effects of AMPR-11 activity on mouse plasma, human serum, low-density lipoprotein (LDL), high-density lipoprotein (HDL), and mouse RBCs. AMPR-11 was incubated with blood components for the indicated times (0, 5, 15, 30, 45, 60, or 75 min) and then with P. aeruginosa or S. aureus for 1 h. Antimicrobial activity was measured with CFU assay. Data represent means ± the SD.

As shown in Fig. 2E, AMPR-11 has fast-acting antimicrobial activity *in vitro*. Therefore, we examined whether this fast-acting property is demonstrated in the murine model of sepsis. Mice infected with S. aureus were administered PBS or AMPR-11 immediately after infection, after which blood was collected from the tail vein at each time described in Fig. 5A. Consistent with the results in Fig. 2, AMPR-11 rapidly decreased the bacterial load in blood (Fig. 5A).

Next, we investigated whether the following blood components affect the antimicrobial activity of AMPR-11: mouse plasma, human serum, human low-density lipoprotein (LDL), human high-density lipoprotein (HDL), bovine serum albumin, and mouse red blood cells (RBCs). AMPR-11 incubated with mouse plasma or human serum showed decreased antimicrobial activity in both P. aeruginosa and S. aureus depending on incubation time (Fig. 5B), and its functional half-life was approximately 50 min in plasma and 40 min in serum. Interestingly, AMPR-11 activity was not inhibited by RBCs, LDL, HDL, or albumin (Fig. 5C), indicating that AMPR-11 does not interact with those factors.

### Efficacy of AMPR-11 in a murine model of sepsis caused by MDR bacteria.

**FIG 6:**
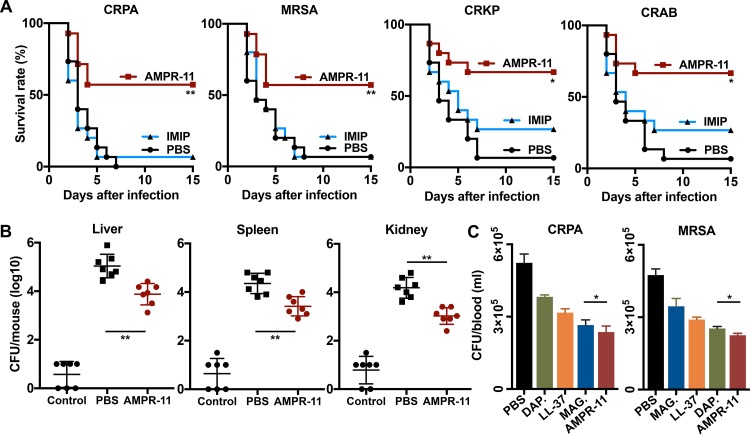
Antimicrobial activity of AMPR-11 in the murine model of sepsis with MDR bacteria. (A) Survival rates in mice infected with MRSA, clinically isolated carbapenem-resistant Gram-negative bacteria (P. aeruginosa [CRPA], K. pneumoniae [CRKP], or A. baumannii [CRAB]). Each group was treated with PBS, imipenem (10 mg/kg with intraperitoneal administration, four times q12h), or AMPR-11 (10 mg/kg with intravenous administration, single dose). Survival rates were calculated with 15 mice/group. (B) CFU assay of liver, spleen, or kidney from mice infected with MDR bacteria. After infection with MRSA, mice were administered PBS or AMPR-11. (C) Comparison of antimicrobial activity of AMPR-11, magainin 2, LL-37, or daptomycin in blood. Mice were infected with CRPA or MRSA, and then each peptide was intravenously administered. The CFU counts of the blood samples from the tail vein were calculated. Data represent means ± the SD. *, *P* ≤ 0.05; **, *P* ≤ 0.01 (by two-way ANOVA). DAP, daptomycin; MAG, magainin 2; IMIP, imipenem.

We next evaluated the efficacy of AMPR-11 in the murine model of sepsis caused by MDR bacteria, including MRSA and carbapenem-resistant Gram-negative bacteria (P. aeruginosa [CRPA], K. pneumoniae [CRKP], and A. baumannii [CRAB]), which were clinically isolated from sputum at Korea University Hospital. As expected, a single dose (10 mg/kg) of AMPR-11 increased the survival rate by >60% in all MDR bacteria (Fig. 6A), with an additional positive outcome of decrease in bacterial load in the liver, spleen, and kidney (Fig. 6B). However, imipenem was of little effectiveness in this experiment. To compare the antimicrobial activity of AMPR-11 *in vivo* with those of well-known AMPs (magainin 2, LL-37, and daptomycin), mice were infected with MRSA or CRPA, and the peptides were administered after 1 h of infection. Blood samples were collected from the tail vein at 1 h after peptide administration. The antimicrobial activity of AMPR-11 in blood was statistically better than that of magainin 2, LL-37, or daptomycin; in contrast to magainin 2 and daptomycin, AMPR-11 demonstrated no bacterial specificity (Fig. 6C). Taken together, these results indicate that AMPR-11 has a broad spectrum of antimicrobial activity in sepsis caused by MDR Gram-negative bacteria or MDR Gram-positive bacteria, has low *in vivo* toxicity, and could be a promising therapeutic option for blood infection diseases (i.e., bacteremia and sepsis).

## DISCUSSION

Antiseptic drug development has not been successful because of the complexity of practical applications. Anti-inflammatory agents such as anti-TNF-α antibody have been evaluated in clinical trials to alleviate inflammation caused by pathogens, but they have not been successful because of different inflammatory mechanisms between humans and experimental animals (34). For this reason, the direction of drug development for sepsis treatment has shifted from inflammatory response to organ dysfunction and disruption of the immune response (35). However, antibiotic treatment is still the inevitable first-line therapy for sepsis treatment.

There are many obstacles for development of new agents to cure sepsis. One of these is that a lack of investment returns in antibiotics development has impeded the discovery of a new class of antibiotics (7). To overcome this hurdle, AMPs derived from host defense protein have been proposed. Unfortunately, low efficacy and high toxicity in systemic application of AMPs have hampered their development against blood infections such as sepsis and bacteremia. For example, intravenous administration of the WLBU2 peptide showed promising efficacy in a mouse sepsis model caused by P. aeruginosa; however, a single dose (16 mg/kg) of WLBU2 peptide killed the mice within 30 min (36). Although orally available talactoferrin was developed against severe sepsis and evaluated in clinical trials, its efficacy was not supported in phase II/III trials. Based on the assumption that the gastrointestinal tracts of such patients were not functionally active, oral uptake of talactoferrin might be not appropriate for sepsis treatment (37). In the present study, we evaluated the efficacy of intravenously administered AMPR-11 and showed it to have low *in vivo* toxicity with a broad spectrum of antimicrobial activity, regardless of bacterial species, including MDR strains.

The superior antimicrobial activity of AMPR-11 in a murine model of sepsis could be explained by several mechanisms. First, the fast-acting activity of AMPR-11 against bacteria might compensate for its short functional half-life. AMPR-11 can kill bacteria within 10 min (Fig. 2E), and its *in vitro* functional half-life in serum or plasma is around 40 or 50 min (Fig. 5B). This functional half-life even shorter *in vivo* (Fig. 5A), possibly due to renal clearance. We speculate that the decrease in half-life caused by renal clearance can be overcome by modification of AMPR-11 with PEGylation or Fc conjugation (38). Second, AMPR-11 did not interact with albumins or lipoproteins, which decrease the activity of AMPs (39). This propensity would increase its activity in conjunction with its fast-acting property in a murine model of sepsis. Since AMPR-11 activity gradually decreased due to unknown factors in serum or plasma, it needs to be further studied to identify its inhibitory factors in blood and to improve its efficacy. For example, it would be worth examining the interaction of AMPR-11 with the serpin superfamily (e.g., α1-antitrypsin and α1-antichymotrypsin) or α2-macroglobulin, which modulate AMP activity (40, 41). Third, AMPR-11 interaction with lipid A could provide an additional effect on mouse survival. Indeed, some AMPs have the ability to interact with endotoxins such as lipopolysaccharide, alleviating systemic inflammation (42). It has been reported that positively charged amino acids in the AMP (R, K, and H) can neutralize negatively charged lipid A, and the hydrophobic region of AMP can interact with acyl chains of lipid A (43). AMPR-11 contains two positively charged amino acids (K58 and R78) and a hydrophobic region (F70 to I77), which might be important for interacting with the negatively charged head group and hydrophobic acyl chains of lipid A, respectively. However, further study is needed to evaluate the decrease of lipid A toxicity caused by interaction with AMPR-11. Fourth, even though the MBC value of AMPR-11 is higher than that of other AMPs or chemical antibiotics, its activity in the murine model of sepsis was very effective. We injected 10 mg/kg of AMPR-11 to mice (28 to 30 g), in which the blood volume of mice was approximately 1.5 to 2 ml. This indicates that the concentration of AMPR-11 is in the range of 150 to 200 μg/ml, which is higher than the MBC value determined in this study (∼100 μg/ml). In contrast to AMPR-11, other AMPs with very low MBC values must be present at much higher concentration to achieve the same effectiveness *in vivo* (36, 44). Therefore, development of novel AMP should take into account multiple assessment criteria such as bactericidal activity, bacteriostatic activity, interaction with blood components, proteolysis, and toxin binding property, which can potentially inactivate AMP. Because the antimicrobial activity of AMP determined from the *in vitro* assay, such as MIC or MBC, is not proportional to *in vivo* efficacy, development of a new *in vitro* screening assay system might be required for effective AMP discovery *in vivo*.

In the present study, we suggest that AMPR-11 has advantages over chemical antibiotics. First, since AMPR-11 has a broad spectrum of antimicrobial activity against Gram-negative bacteria and Gram-positive bacteria, including MDR strains that are known to cause sepsis, it could treat bacterial species in patients who have symptoms of sepsis before identification of the species from patient blood. Second, in terms of AMP resistance, Gabriel et al. reported experimental evolution of resistance to pexiganan, which was previously evaluated for DFI. This experimental resistance was acquired over 600 to 700 generations (45). Indeed, resistance to pexiganan has not yet been detected in clinical trials (46), in contrast to the resistance to chemical antibiotics often detected during clinical trials (47). This implies that resistance against AMP might confer a higher fitness cost to bacteria compared to chemical antibiotics. Third, AMPR-11 might be degraded before excretion like other peptide-based medicines, indicating no concerns of environmental pollution. Indeed, chemical antibiotics have been utilized extensively in the livestock and agriculture industries, resulting in an increase in MDR bacteria. For this reason, there is a concern that the antibiotics used for nonhuman purposes can induce the emergence of MDR bacteria, which in turn infect humans (48).

AMPR-11 could be combined with other potential antibiotics. In contrast to Gram-positive bacteria, Gram-negative bacteria have efflux pumps to remove antibiotics from the bacterial cytoplasm, and this has frequently been reported as a mechanism to gain antibiotic resistance in Gram-negative bacteria (49). Cirioni et al. reported the synergistic combination of magainin 2 and rifampin against MDR P. aeruginosa, suggesting that membranolytic AMP could allow antibiotics to access the intracellular space of bacteria. However, its synergistic effect was shown only with an aminoglycoside antibiotic, in the case of the P5 peptide. As a standard treatment protocol for sepsis based on guidelines, empirical antibiotic combinations should be administered before bacterial characterization; thus, AMPR-11 cannot be administered alone but should be administered with an empirical antibiotic combination. Moreover, AMPR-11 showed good efficacy even as a single dose; therefore, it would be valuable to evaluate the effect of multiple doses of AMPR-11, which might increase the antimicrobial efficacy in the murine model of sepsis model. For this reason, antibiotic combination with AMPR-11 is a promising therapeutic strategy and needs to be further studied.

In this study, we used a single dose (10 mg/kg) of AMPR-11 to evaluate its efficacy in a murine model of sepsis induced by 2 × 10^7^ to 8 × 10^8^ CFU of bacteria, which is commonly used for sepsis models. However, human sepsis is much more sensitive than that of mice in terms of inflammatory responses: 100 CFU/ml in blood induce sepsis in humans (50). The blood volume of the mice we used in this study (28 to 30 g) was approximately 1.5 to 2 ml, indicating that we injected bacteria at a rate of 2 × 10^5^ to 8 × 10^7^ times higher than the pathological concentration of bacteria in humans. Since AMP activity is sensitive to the lipid-AMP ratio for penetrating bacteria (51), the amount of AMPR-11 could be decreased in clinical trial. This would significantly decrease the price of AMPR-11, thereby decreasing manufacturing cost, which is a limitation of AMPs compared to chemical antibiotics. In conclusion, we suggest AMPR-11 as a promising therapeutic option for sepsis/bacteremia caused by MDR bacteria. Considering that the ideal AMP for sepsis will eradicate bacteria regardless of species and the presence of MDR and should have low *in vivo* toxicity, AMPR-11 could be a promising antimicrobial candidate for MDR bacteria causing sepsis/bacteremia.

## MATERIALS AND METHODS

### Chemicals.

E. coli total lipid extract, egg l-α-phosphatidylcholine (PC), egg l-α-phosphatidylglycerol (PG), brain l-α-phosphatidylserine (PS), egg l-α-phosphatidylethanolamine (PE), egg l-α-phosphatidic acid (PA), liver l-α-phosphatidylinositol (PI), lipid A, cardiolipin (CL), and TF-CHOL were purchased from Avanti Polar Lipids (Alabaster, AL). Human serum, human LDL, human HDL, bovine serum albumin, and all chemicals were purchased from Sigma-Aldrich (St. Louis, MO). Romo1 peptide with or without TAMRA and LL-37 was chemically synthesized by GL Biochem (Shanghai, China); all other peptides were synthesized by ANYGEN (Gwangju, South Korea) and purified by high-performance liquid chromatography.

### Bacterial strains.

EIEC (NCCP 13719), S. pneumoniae (NCCP 14585), vancomycin-resistant S. aureus (NCCP 15872), and vancomycin-resistant E. faecium (NCCP 11522) were purchased from the National Culture Collection for Pathogens (NCCP; Cheongju, South Korea). P. aeruginosa (ATCC 27853), S. aureus (ATCC 29213), K. pneumoniae (ATCC 13883), A. baumannii (ATCC 19606), Bacillus subtilis (ATCC 6633), E. faecium (ATCC 19434), Streptomyces sindenensis (ATCC 12392), Enterococcus faecalis (ATCC 19433), E. coli (ATCC 25922), Enterobacter aerogenes (ATCC 13048), and MRSA (ATCC 33591) were purchased from American Type Culture Collection (ATCC; Gaithersburg, MD). CRPA, CRKP, and CRAB were clinically isolated in Korea University Hospital (Institutional Review Board, no. 2015AN0129). CRPA is resistant to piperacillin, piperacillin-tazobactam, ceftazidime, imipenem, meropenem, gentamicin, amikacin, and ciprofloxacin. CRAB is resistant to piperacillin, piperacillin-tazobactam, cefepime, ceftazidime, imipenem, meropenem, gentamicin, amikacin, and ciprofloxacin. CRKP is resistant to piperacillin-tazobactam, cefepime, ceftazidime, imipenem, gentamicin, and ciprofloxacin. GFP-expressing EIEC were transformed with AcGFP1-C1 plasmid. All strains were stored at –80°C in 50% (vol/vol) glycerol and 50% (vol/vol) Luria-Bertani (LB) or tryptic soy (TS) broth, grown on LB or TS plates, and aerated at 37°C.

### Bacteria invasion of HeLa cells.

Bacteria invasion experiments were performed as previously described with minor modifications (52). Briefly, HeLa cells were maintained as monolayers in minimum essential Eagle medium (MEM) with 10% fetal bovine serum in a 37°C, 5% CO_2_ incubator. HeLa cells were seeded at 1.5 × 10^5^ cells per well and incubated for 24 h. EIEC was diluted with MEM and infected the HeLa cells with 500 μl of medium containing 5 × 10^2^ CFU without antibiotics for 4 h at 37°C. The plates were washed three times with PBS and incubated with 100 μg/ml gentamicin in medium for 1 h. The HeLa cells were washed and lysed with 200 μl of 0.1% Triton X-100 in PBS for 30 min on ice. Serial dilutions of cell lysate were plated on LB agar and incubated overnight to determine the number of bacterial colonies. Romo1 double-stranded small interfering RNA (siRNA) oligonucleotides were synthesized by Bioneer (Daejeon, South Korea). The sequences were 5-TTCTCCGAACGTGTCACGT-3 for control siRNA and 5-GGGCTTCGTGATGGGTTG-3 and 5-AACCATGATGCAGAGTGGCGGCACCTT-3 for Romo1 siRNA-1 and Romo1 siRNA-2, respectively. These were transfected using Lipofectamine 3000.

### Bactericidal assay.

The minimum bactericidal concentration was determined as previously described with minor modifications (29). Briefly, bacteria (5 × 10^5^ CFU/ml) in 10 mM phosphate buffer (pH 7.4) with a 1% volume of TS broth were incubated with various peptide dilutions in a 96-well plate. After incubation for 1 h, samples were placed on TS agar plates and incubated overnight at 37°C. The peptide concentration at which no colonies were identified on the plate was determined to be the MBC.

### Flow cytometric assay.

Flow cytometric assay to measure the efflux of GFP from GFP-expressing EIEC was performed using a FACS Canto II (Biosciences, CA). GFP-expressing EIEC with 100 nM AMPR-11 was incubated in PBS at room temperature for 10 min. The number of internalized EIEC in HeLa cells (pcDNA 3.1 or pcDNA 3.1-Flag-Romo1/Control siRNA, Romo1 siRNA-1, or Romo1 siRNA-2) was measured using CFDA-stained EIEC. The data were measured and analyzed with FlowJo software (Tree Star, Inc., Ashland, OR).

### Scanning electron microscopy.

Portions (10^9^ CFU) of bacteria incubated with or without peptides (250 μg) were fixed with 2.5% glutaraldehyde in 0.1 M phosphate buffer (pH 7.4) for 2 h at 25°C and centrifuged at 1,000 rpm. After washing the pellet twice for 20 min, samples were postfixed with 2% osmium tetroxide for 2 h and rinsed with distilled water for 5 min. After standard dehydration in ethanol (60, 70, 80, 90, 95, and 100%), samples were freeze-dried (ES-2030; Hitachi, Ltd., Tokyo, Japan), attached to a stub, and coated with platinum using an ion sputter (HitachiE-1045). The images were observed using a Hitachi S-4700 scanning electron microscope.

### Transmission electron microscopy.

Portions (10^9^ CFU) of bacteria incubated with or without Romo1 (250 μg) were fixed with 2% paraformaldehyde and 2.5% glutaraldehyde in 0.1 M phosphate buffer (pH 7.4) and centrifuged at 1,000 rpm. Samples were postfixed with 2% osmium tetroxide for 2 h and rinsed with distilled water for 5 min. After standard dehydration in ethanol (60, 70, 80, 90, 95, and 100%), samples were infiltrated and embedded in propylene oxide/Epon mixture. Thin sections (1 μm) were obtained using an ultramicrotome (UC7; Leica, Vienna, Austria) on a grid with toluidine blue staining. The images were observed using a Hitachi H-7650 transmission electron microscope with 80 kV acceleration voltage.

### Giant unilamellar vesicle preparation.

Portions (10 μl) of E. coli total lipid extract with 0.5% TF-CHOL were dissolved in chloroform (5 mg/ml) and dried on indium tin oxide-coated glass at 50°C. The chamber was filled with 300 mM sorbitol, and GUVs were produced by 2 V peak-to-peak and 8 Hz for 120 min at 36°C using Vesicle Prep Pro (Nanion Technologies GmbH, Munich, Germany). GUV images were acquired with a Zeiss LSM 700 and analyzed using ZEN 2 software (Zeiss GmbH, Jena, Germany).

### Large unilamellar vesicle preparation.

Portions (10 μl) of the lipid mixture of E. coli total lipid extract were dried and rehydrated with 50 mM CF, 100 mM sucrose, and 5 mM HEPES/KOH (pH 7.4). Multilamellar liposomal suspensions were extruded with a 0.1-μm polycarbonate membrane using an Avanti Mini Extruder and purified by a PD-10 column (GE Healthcare, Buckinghamshire, United Kingdom) as previously described. The peptides were added to LUVs, and CF leakage was measured using Fluroskan Ascent FL (Thermo Labsystems, UK) in external buffer (150 mM KCl and 10 mM HEPES/Tris; pH 7). CF leakage was calculated using the following formula: CF leakage (%) = 100 × (*F* – *F*_0_)/(*F*_max_ – *F*_0_), where *F* is the measured fluorescence intensity, *F*_0_ is the basal LUV fluorescence intensity, and *F*_max_ is the fluorescence intensity of LUVs treated with 0.2% Triton X-100.

### Protein lipid overlay assay.

The experiment was performed as previously described (53) with minor modifications. The lipid of PC, PG, PS, PE, PA, PI, lipid A, or CL was diluted and placed on the supported nitrocellulose membrane at 20 pmol and dried at room temperature. The membrane was blocked with Tris-buffered saline-Tween 20 (TBS-Tween 20, 50 mM Tris-HCl, 150 mM NaCl, and 0.1% Tween 20) containing 0.2% fatty acid-free bovine albumin for 1 h. After several washes in TBS, the membrane was incubated with TAMRA-AMPR-11 at 2 μg/ml in blocking solution for 2 h. After several washes with TBS-Tween 20, the fluorescent signal was detected using a fluorescent image scanner (Typhoon FLA 9500).

### Circular dichroism.

The secondary structure of the AMPR-11 was examined by circular dichroism (CD) spectroscopy using Chirascan (Applied Photophysics, Ltd., Leatherhead, UK). The peptide concentration was 100 μM in a 1-mm-path length quartz cuvette with distilled water or 50% HFIP. The CD spectra were corrected for background scattering by subtracting a buffer-only spectrum measured without peptide. The samples were recorded at 25°C between 180 and 260 nm.

### Cell viability assay.

The viabilities of the mammalian cells (HeLa cells, HEK293 cells, and HUVECs) were determined using the MTT [3-(4,5-dimethylthiazol-2-yl)-2,5-diphenyltetrazolium bromide] assay. A portion (1 × 10^4^) cells was seeded on a 96-well plate, followed by incubation with AMPR-11, daptomycin, or magainin 2. After incubation for 2 h, the cells were incubated with MTT solution at 2 mg/ml in PBS for 1 h. The medium was removed, and the purple formazan crystals were solubilized by dimethyl sulfoxide. The plate was gently tapped, and the optical density (OD) absorbance was measured at 550 nm.

### *In vitro* hemolysis assay.

Mouse RBCs were rinsed several times with PBS and centrifuged for 10 min at 2,000 rpm. The hematocrit at 1% (vol/vol) was resuspended in PBS, and aliquots of 100 μl of RBC solution were incubated with 2-fold serial dilutions of AMPR-11, daptomycin, magainin 2, or melittin. After incubating for 1 h, the cells were pelleted at 1,000 × *g* for 10 min, and the absorbance of the supernatant was measured at 570 nm. The OD of the control was that of PBS (blank), and 1% Triton X-100 was used as the positive control.

### Murine model of sepsis.

Male C57BL/6N mice (10 weeks old; weight, 28 to 30 g) were obtained from Orient Bio, Inc. (Gyeonggi-do, South Korea). After 1 week of quarantine, all mice were housed in an animal biosafety level 2 (ABSL-2) facility with free access to food and water on a 12-h light and 12-h dark cycle. Forty-five male mice were randomly divided into three groups of equal size. Each bacterium was intravenously injected using a 1-ml insulin syringe 30 G (P. aeruginosa, 2 × 10^7^ CFU; S. aureus, 1 × 10^8^ CFU; A. baumannii, 4 × 10^8^ CFU; *K. pneumonia*, 8 × 10^8^ CFU; CRPA, 8 × 10^7^ CFU; MRSA, 3 × 10^8^ CFU; CRAB, 5 × 10^8^ CFU; CRKP, 8 × 10^8^ CFU). The treatment group of AMPR-11 (10 mg/kg) was intravenously treated after 1 h. The imipenem treatment group was intraperitoneally injected four times after 24 h. The volume of each intravenous or intraperitoneal injection was no more than 150 μl. Deaths were assessed every day for 15 days. For CFU determination, blood samples were collected from the mouse tail vein, diluted, and placed on an LB agar plate after treatment with AMPR-11 or another antibiotic (imipenem, daptomycin, magainin 2, or LL-37) for 1 h. Assessment of antimicrobial activity of AMPR-11 was conducted with 75% PBS and 25% mouse plasma, which were separated using EDTA separation, or with human serum (H4522; Sigma-Aldrich) as previously described with minor modifications (54). For CFU determination in organs, the isolated organs (liver, spleen, and kidney) were homogenized with a glass-tissue grinder in ice-cold PBS. The diluted samples were plated on an LB agar plate. The animal experiments were approved by the Institutional Animal Care and Use Committee (IACUC) of Korea University College of Medicine (KOREA-2016-0256, KOREA-2018-0022, and KOREA-2018-0151).
