# Supplementary figures and images for: Romo1-Derived Antimicrobial Peptide Is a New Antimicrobial Agent against Multidrug-Resistant Bacteria in a Murine Model of Sepsis
Source: mBio. 2020 Apr 14;11(2):e03258-19. doi: 10.1128/mBio.03258-19 (PMC7157825; doi:10.1128/mBio.03258-19)

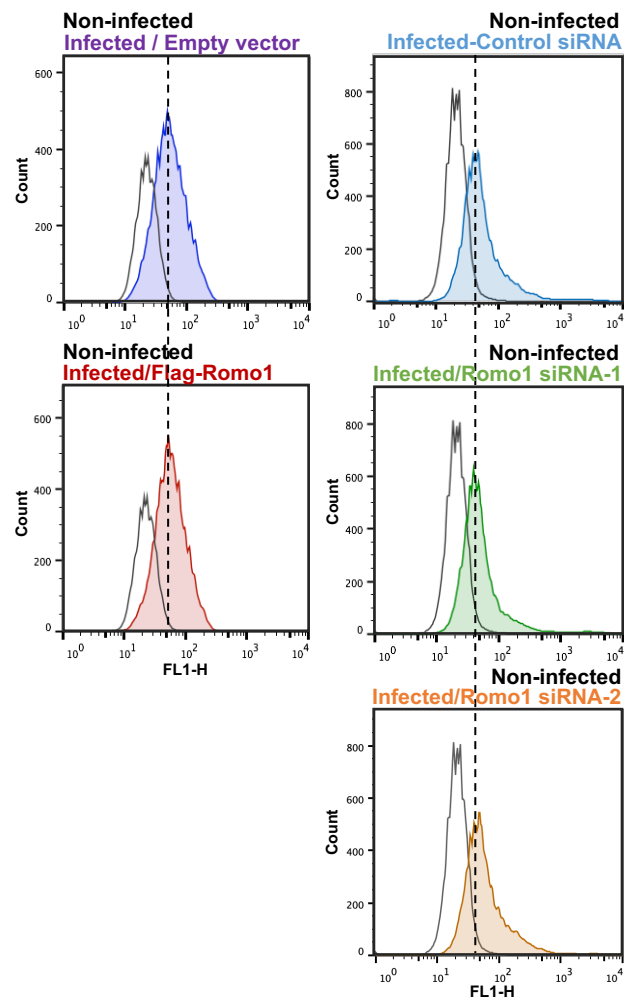

**FIG S1**

Supplement: FIG S1 [file mBio.03258-19-sf001.pdf]
