# Supplementary material for: Romo1-Derived Antimicrobial Peptide Is a New Antimicrobial Agent against Multidrug-Resistant Bacteria in a Murine Model of Sepsis
Source: mBio. 2020 Apr 14;11(2):e03258-19. doi: 10.1128/mBio.03258-19 (PMC7157825; doi:10.1128/mBio.03258-19)
Supplement: TABLE S1 [file mBio.03258-19-st001.docx]

**Table S1** MBC determination of AMPR-11 in multiple bacterial species.

| **Group** | **Bacteria** | **AMPR-11**  **MBC value**  **(µg/ml)** |
| --- | --- | --- |
| **Gram (+)** | *S. aureus* | 100 |
|  | *B. subtilis* | 90 |
|  | *E. faecium* | 85 |
|  | *S. sindenensis* | 95 |
|  | *E. faecalis* | 85 |
|  | *S. pneumoniae* | 100 |
| **Gram (-)** | *E. coli* | 85 |
|  | *P. aeruginosa* | 100 |
|  | *K. pneumoniae* | 100 |
|  | *A. baumannii* | 100 |
|  | *E. aerogenes* | 90 |
| **MDR** | Methicillin-resistant *S. aureus* | 100 |
|  | Carbapenem-resistant *P. aeruginosa* | 110 |
|  | Carbapenem-resistant *A. baumannii* | 110 |
|  | Carbapenem-resistant *K. pneumoniae* | 100 |
|  | Vancomycin-resistant *S. aureus* | 120 |
|  | Vancomycin-resistant *E. faecium* | 100 |
